# Multiple Measures Reveal Antiretroviral Adherence Successes and Challenges in HIV-Infected Ugandan Children

**DOI:** 10.1371/journal.pone.0036737

**Published:** 2012-05-09

**Authors:** Jessica E. Haberer, Julius Kiwanuka, Denis Nansera, Kathleen Ragland, Claude Mellins, David R. Bangsberg

**Affiliations:** 1 Center for Global Health, Massachusetts General Hospital, Boston, Massachusetts, United States of America; 2 Harvard Medical School, Boston, Massachusetts, United States of America; 3 Department of Paediatrics, Mbarara University of Science and Technology, Mbarara, Uganda; 4 Departments of Psychiatry and Sociomedical Sciences, Columbia University, New York, New York, United States of America; Vanderbilt University, United States of America

## Abstract

**Background:**

Adherence to HIV antiretroviral therapy (ART) among children in developing settings is poorly understood.

**Methodology/Principal Findings:**

To understand the level, distribution, and correlates of ART adherence behavior, we prospectively determined monthly ART adherence through multiple measures and six-monthly HIV RNA levels among 121 Ugandan children aged 2–10 years for one year. Median adherence levels were 100% by three-day recall, 97.4% by 30-day visual analog scale, 97.3% by unannounced pill count/liquid formulation weights, and 96.3% by medication event monitors (MEMS). Interruptions in MEMS adherence of ≥48 hours were seen in 57.0% of children; 36.3% had detectable HIV RNA at one year. Only MEMS correlated significantly with HIV RNA levels (r = −0.25, p = 0.04). Multivariable regression found the following to be associated with <90% MEMS adherence: hospitalization of child (adjusted odds ratio [AOR] 3.0, 95% confidence interval [CI] 1.6–5.5; p = 0.001), liquid formulation use (AOR 1.4, 95%CI 1.0–2.0; p = 0.04), and caregiver’s alcohol use (AOR 3.1, 95%CI 1.8–5.2; p<0.0001). Child’s use of co-trimoxazole (AOR 0.5, 95%CI 0.4–0.9; p = 0.009), caregiver’s use of ART (AOR 0.6, 95%CI 0.4–0.9; p = 0.03), possible caregiver depression (AOR 0.6, 95%CI 0.4–0.8; p = 0.001), and caregiver feeling ashamed of child’s HIV status (AOR 0.5, 95%CI 0.3–0.6; p<0.0001) were protective against <90% MEMS adherence. Change in drug manufacturer (AOR 4.1, 95%CI 1.5–11.5; p = 0.009) and caregiver’s alcohol use (AOR 5.5, 95%CI 2.8–10.7; p<0.0001) were associated with ≥48-hour interruptions by MEMS, while second-line ART (AOR 0.3, 95%CI 0.1–0.99; p = 0.049) and increasing assets (AOR 0.7, 95%CI 0.6–0.9; p = 0.0007) were protective against these interruptions.

**Conclusions/Significance:**

Adherence success depends on a well-established medication taking routine, including caregiver support and adequate education on medication changes. Caregiver-reported depression and shame may reflect fear of poor outcomes, functioning as motivation for the child to adhere. Further research is needed to better understand and build on these key influential factors for adherence intervention development.

## Introduction

As of the end of 2009, an estimated 2.5 million children were living with HIV/AIDS globally, and 354,000 were receiving antiretroviral therapy (ART) [1]. Studies to date on adherence to ART among pediatric populations in developing settings have shown mixed results with adherence ranging from 49% to 100% [2]. Factors related to family structure [3], [4], socioeconomic status [3], [5], disclosure [5], [6], [7], [8], hospitalization [8] and medication routine and/or regimen [4], [5], [9] have all been significantly associated with ART adherence. Most studies have relied on caregiver-report [3], [4], [5], [9], [10], [11], [12], although some studies have used objective measures such as pill counts [5], [8], [12], [13], pharmacy records [14], therapeutic drug monitoring [15], directly observed therapy [16], and electronic monitoring [5], [7], [17]. Few have compared multiple adherence measures to HIV RNA [5], [17]. Variations in measures and in adherence estimates call for a more comprehensive and accurate assessment of adherence to ART in this population in order to develop evidence-based interventions for promoting sustained adherence.

This observational study prospectively measured adherence using caregiver report, unannounced pill count/weight of liquid formulations, and medication event monitors (MEMS) in 121 children taking ART in rural, southwestern Uganda. Following an adaptation of a previously published conceptual framework for understanding pediatric adherence behavior [18], associations were examined among adherence and various child, antiretroviral (ARV) regimen, caregiver, and household/community characteristics. This study adds to the current literature by comparing multiple adherence measures, including HIV RNA, which were collected over one year in a relatively large sample receiving routine clinical care. The primary objectives of the study were to determine the levels, distribution, and correlates of ART adherence among children in a developing setting. A secondary objective was to determine which measure correlates best with HIV RNA levels and is most feasible in a developing setting.

## Methods

### Ethical Statement

This study was registered with Clinical Trials (NCT00868257) and was approved by institutional review boards at the Mbarara University of Science and Technology, the Uganda National Council for Science and Technology, and Partners Healthcare (see Protocol S1). Written, informed consent was obtained from all caregivers, and verbal assent was obtained from children at least seven years old.

### Study Setting and Population

The participants in this study were recruited from the Children’s HIV/AIDS Care Clinic at the Mbarara University Regional Referral Hospital, which serves a largely rural population in southwestern Uganda. Approximately 700 children are actively followed in the clinic with 520 taking ART, which is provided free-of-charge and according to Ugandan National Guidelines [19]. No changes in the Ugandan National Guidelines occurred during the course of the study. All ARVs were dispensed by the hospital pharmacy. Children were eligible for this study if they were aged 2–10 years old, HIV-positive, already taking ART or initiating ART at enrollment, and residing within 20 km of the clinic, beyond which logistics of data collection would not have been possible. Children aged 2–10 years were targeted because this age range represents a large proportion of HIV-infected children in care [20], [21], and the requirements for care within this age range are relatively similar and distinct from those for infants and adolescents [18]. The only exclusion criterion was residence at a boarding school, which would have presented substantially different circumstances of adherence behavior compared to children living at home. All eligible participants identified between July 2008 and February 2009 were recruited for the study; data were collected between July 2008 and March 2010.

### Adherence Measures

Adherence was measured monthly by three methods: 1) caregiver report, using three-day recall and 30-day visual analog scale [VAS]); 2) unannounced pill count/weight of liquid formulations at the child’s home, the timing of which was variable within a two to six week period to avoid predictability; and 3) the medication event monitoring system (MEMS), which recorded bottle openings for both pill and liquid formulations. Adherence by pill count was calculated as (the number of pills dispensed – number of pills counted)/(the number of pills expected to be taken) in the previous month. A similar calculation was used for liquid formulations, substituting grams for pills; the weight of the medication bottle was subtracted from measured weights to determine the weight of the medication present. MEMS adherence was calculated as (the number of events/the number of expected events) * 100. The timing of the opening was not considered in the calculation, as the contribution of dose timing to viral suppression is controversial [17], [22] and MEMS events may or may not correlate precisely with medication ingestion (i.e. participants may remove multiple doses at one opening or open the bottle without removing medication). MEMS data was also reviewed for ≥48-hour interruptions, which have been shown to predict virologic failure and resistance to non-nucleoside reverse transcriptase inhibitor (NNRTI)-based therapy [23], [24].

### Child, Arv Regimen, Caregiver, and Household/Community Characteristics

A research assistant administered a structured interview to participant caregivers at baseline and every three months over the one-year study period. All questions were translated into the local language (Runyankole) and back translated to ensure proper interpretation. The interview covered various socio-demographic, behavioral, and clinical factors with potential to influence adherence behavior (see Table 1), and included the following standardized measures: 1) Caregiver depressive symptoms were assessed using the Hopkins Symptom Checklist Depression Scale (with a cut off for possible depression defined as a score ≥1.75), which was found to have criterion validity with a grief syndrome in neighboring Rwanda [25]; 2) Physical symptoms were measured with a scale drawn from the AIDS Clinical Trials Group symptom survey [26]; 3) Quality of life was examined in caregivers by the physical and mental health component summary scales of the Short-Form 12 version 2 (SF-12v2; standardized to a mean of 50 based on the US population) [27] and in children aged five and older by the Pediatric AIDS Clinical Trials Group (PACTG) Quality of Life Assessment, which includes general health perception, symptom distress, psychological status, and physical function domains (each standardized to a range of 0–100) [28]. ARV regimen characteristics were recorded during the monthly unannounced pill counts/liquid formulation weights.

**Table 1:** Characteristics of the child, regimen, caregiver, and household/community.

Child characteristics	N*		Caregiver characteristics	N*	
Median age	120	5.2 years (IQR 3.6–7.2)	Biologic mother or father	119	84 (70.6%)
Age 5 years or older	120	65 (54.2%)	Female	121	104 (86.0%)
Female	118	56 (47.5%)	Change in caregiver	121	6 (5.0%)
ART experience at enrollment	121	105 (86.8%)	HIV infected	120	89 (74.2%)
Median duration of ART	85	26 months (IQR 11.5–41.8)	Taking ART	119	32 (26.7%)
Median CD4%	107	40% (IQR 28%–48%)	Greater than primary school education	121	38 (31.4%)
HIV RNA <400 copies/ml	91	53 (58.2%)	Employed	121	75 (62.0%)
ART initiated at enrollment	121	16 (13.2%)	Possible depression^3^	120	30 (25.0%)
Median CD4%	14	27% (16%–32%)	SF-12v2: Physical composite score	117	53.8 (IQR 48.9–55.9)
Hospitalized in the past three months	111	6 (5.4%)	SF-12v2: Mental composite score	117	49.0 (IQR 41.6–54.7)
Received PMTCT	89	4 (4.5%)	Any alcohol use	120	11 (9.2%)
Physical symptoms reported^1^	116	41 (35.3%)	Heavy alcohol use^4^	120	4 (3.3%)
Median # of symptoms reported, if any	41	2 (IQR 1–3)	Help available with the child	119	75 (63.0%)
Attending (pre)school, if aged 5 or older	63	41 (65.1%)	More than one person gives ART to the child	117	34 (29.1%)
PACTG quality of life			Difficulty telling others about the child’s HIV	116	84 (72.4%)
Median general health perception	104	81.0 (IQR 66.7–88.9)	Ashamed of the child’s HIV status	115	69 (60.0%)
Median symptom distress	97	71.4 (IQR 64.3–78.6)	Hides the child’s HIV status	116	80 (69.0%)
Median psychological status	98	96.4 (IQR 89.3–100.0)	**Household/community characteristics**		
Median physical functioning	99	100.0 (IQR 95.8–100.0)	Place to keep ART	100	97 (97.0%)
Use of co-trimoxazole^2^	113	95 (84.1%)	Median asset index	111	0.5 (IQR -1.7–1.4)
Knowledge of HIV status	120	18 (15.0%)	Median minutes traveled to clinic	120	30 (20–60)
**Regimen characteristics**			Median cost of travel to clinic	119	4000 UgSh (2000–7000)^5^
NNRTI backbone	121	114 (94.2%)	Median # of siblings	121	2 (IQR 1–3)
PI backbone	121	7 (5.8%)	Median # of siblings with HIV	121	1 (IQR 0–1)
Liquid formulation used at least once	121	42 (34.7%)	Median # of other children in household	121	0 (IQR 0–2)
Change in ARV manufacturer at least once	121	99 (81.8%)	Median # of other children in household with HIV	121	0 (IQR 0–1)
Change in ARV formulation at least once	121	16 (13.2%)	Disclosure of child’s HIV to the household	115	109 (94.8%)
Use of a 2-drug FDC at least once	121	23 (19.0%)	Disclosure of child’s HIV to the community	115	70 (60.9%)
Use of a 3-drug FDC at least once	121	10 (8.3%)			

* N = sample used for determining each characteristic, IQR = interquartile range, FDC = fixed drug combination, UgSh = Ugandan Shillings.

1 Most common symptoms were cough, weakness/tiredness, and skin problems.

2 Co-trimoxazole was prescribed to all children on enrollment for prevention of opportunistic and other infections regardless of ARV use.

3 The most common and consistently endorsed symptom on this scale was “worrying too much about things”.

4 Defined as >5 drinks (1 glass wine, 333 ml beer, or 40 ml hard liquor).

5 4000 UgSh equals approximately US$1.60.

### Laboratory Tests

National Guidelines at the time of the study recommended CD4 percentages and HIV RNA levels every six months as part of standard clinical care. When performed, these values were matched with adherence data determined at baseline, six months, and/or 12 months, if they were drawn within six weeks before or after the time point. CD4 percentages were measured by standard flow cytometry techniques (FACSCount, BD, New Jersey, USA). HIV RNA levels were determined by the Amplicor HIV-1 Monitor Test (Roche, New Jersey, USA) with a lower limit of detection of 400 copies/ml.

### Analysis Methods

Monthly adherence values were analyzed as continuous variables; averaged (mean) data were determined for each participant and reported as medians for the entire cohort. MEMS data were censored for known device non-use, including periods with a non-functional MEMS cap or where the caregiver reported not using the device (e.g. while traveling). Assuming a standard deviation of 9, the sample size of 121 has >80% power to detect an adherence level of 90.0+/−1.7%.

Nonparametric statistical tests were used, given the non-normal distribution of the data. Wilcoxon signed rank and Chi-square tests were used for bivariable comparisons of continuous and categorical adherence measures, respectively. Agreement among adherence measures and HIV RNA was determined by Spearman correlation at six and twelve months. Adherence data were compared with each other using data from the full one-year study period; comparisons with HIV RNA reflect the month prior to the HIV RNA test.

A principal component analysis was performed to create an asset index serving as a proxy for socio-economic status. This index is based on the methodology of Filmer-Pritchett [29] and includes home ownership, land ownership, electricity, livestock ownership, good transport, and food security.

Predictors of adherence were determined by generalized linear mixed models (SAS proc glimmix), first using univariable regressions on <90% MEMS adherence for the full study period. This level of adherence was chosen because reliable viral suppression in NNRTI-based regimens is unlikely at lower levels of adherence [30], and it is consistent with the levels of adherence assessed in previous studies (85%, 95%, and 100%) [2]. If the p-value was found to be <0.10, the predictor was retained for multivariable analysis. A second multivariable regression was similarly examined for the presence of ≥48 hour interruptions in MEMS adherence data. The following variables were examined (selected from Table 1): child characteristics (age, gender, ART history, enrollment CD4 percentage, hospitalization in the previous three months, presence of physical symptoms, quality of life, attending school, use of co-trimoxazole, knowledge of HIV status); regimen characteristics (use of liquid formulation at least once, change in drug manufacturer or formulation at least once, use of second line ART); caregiver characteristics (biological relationship to the child, change in primary caregiver, HIV status, use of ART, education level, employment status, possible depression, physical and mental health, use of alcohol, more than one caregiver, hiding the child’s HIV status, feeling ashamed and having difficulty telling others about the child’s HIV status); and household characteristics (place to keep ART, time and cost of travel to clinic, number of household members, asset index, having to sell property, disclosure of child’s HIV status within the household and to the community).

All analyses were performed using SAS (version 9.2, Cary, NC, USA). With the exception of the multivariable regression model, statistical significance was set at p<0.05. Missing data were treated as missing.

## Results

### Study Participants

**Figure 1:**
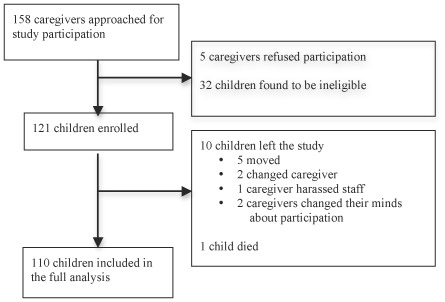
Study participants.

A total of 158 caregivers were approached for participation in this study, of whom five (3.2%) refused participation and 32 (20.3%) were found to be ineligible (Figure 1). The most common reason for ineligibility was residence beyond 20 km from the clinic. The first 121 children eligible for participation were enrolled in the study. The baseline characteristics of the children, ARV regimens, caregivers, and households/communities are detailed in Table 1. Briefly, most children were taking NNRTI-based ART at enrollment and were clinically stable. Most caregivers were the biological mother with limited education and were HIV-infected themselves. Nearly all households were aware of the child’s HIV status, but most children did not know they were HIV-infected.

Ten children left the study (five moved out of the study catchment area, two changed caregivers and the new caregivers were not interested in participating, and two caregivers changed their minds about participating in the study; one child’s caregiver exhibited disruptive behavior), and one child died of diarrheal illness near the end of the study. Data for these 11 children were censored at the time they left the study. When comparing the children who left and did not leave the study, no differences were seen in child, caregiver, or baseline regimen characteristics. Households, however, were significantly smaller in children who left (mean 2.5 versus 4.5 members, p = 0.004) with fewer other children with HIV (mean 0 versus 0.3 children, p = 0<0.001). No children were lost to follow up. No adverse events were reported.

### HIV RNA Levels

**Figure 2:**
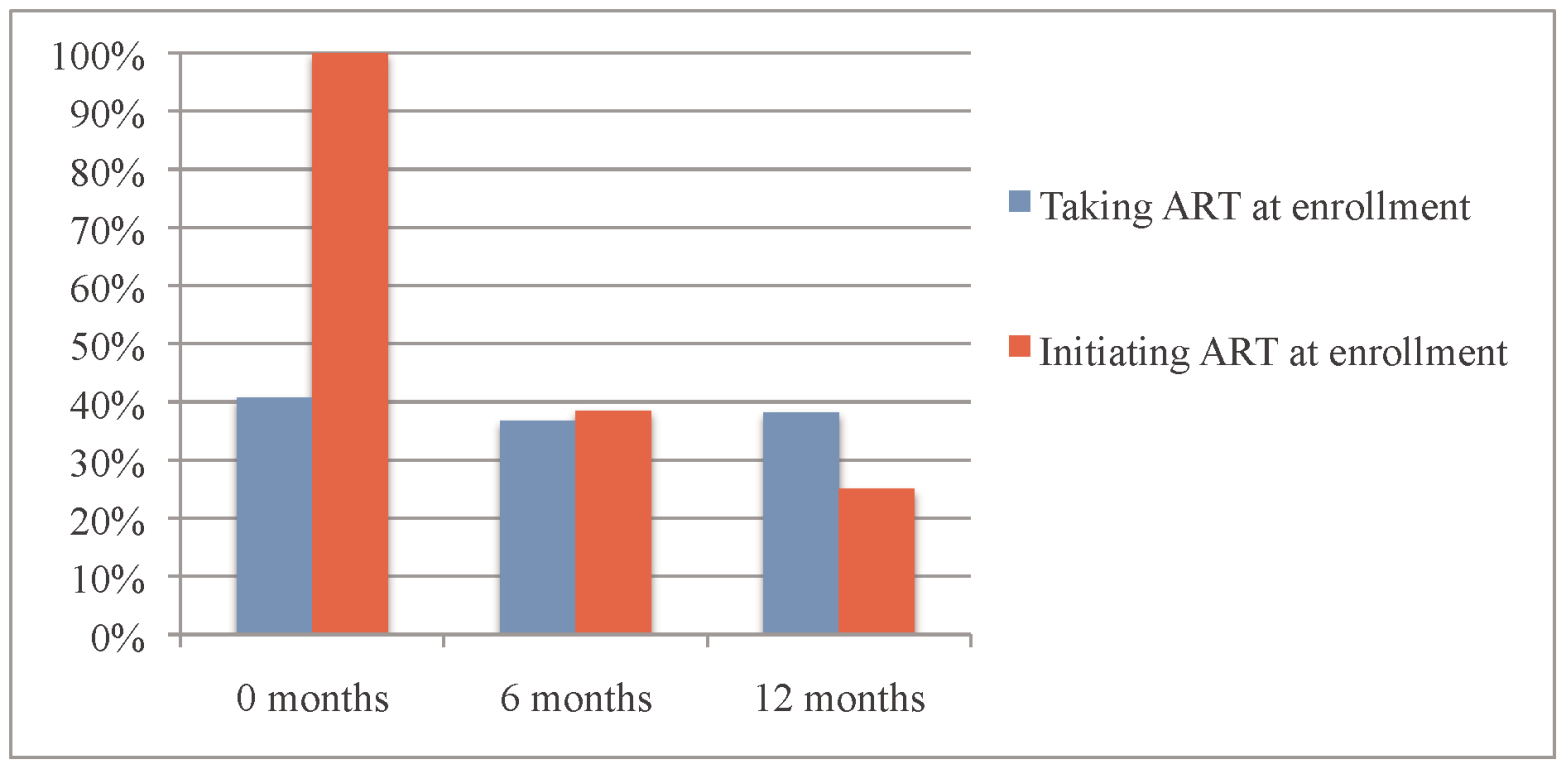
Detectable HIV RNA by ART history at enrollment.

Detectable HIV RNA (defined as >400 copies/ml) is shown in Figure 2. Fewer than 5% of detectable HIV RNA levels were <1,000 copies/ml, making the likelihood of transient, minor elevations, or “blips”, low [31]. Among participants already taking ART at baseline, levels of detectable HIV RNA remained essentially unchanged with 41.8%, 36.7%, and 38.2% at 0, 6, and 12 months, respectively. Among participants initiating ART at baseline, levels of detectable HIV RNA were 100.0%, 38.5% and 25.0% at 0, 6, and 12 months, respectively. HIV RNA values were missing for 18 (14.9%), 35 (28.9%), and 31 (25.6%) participants at 0, 6, and 12 months, respectively. These samples were not drawn due to sporadic variances in routine clinical care and were not systematically missed.

### Median Adherence and Adherence Patterns

Median adherence for the cohort was high by all measures, ranging from 96.3 to 100.0% (Table 2). Midway through the study, hospital pharmacists were found to be taking back and giving out extra medication in efforts to facilitate adherence among their patients. The number of participants affected by their efforts and the extent of the medication manipulation was not recorded, thus preventing adjustment of the unannounced pill counts/liquid weights. MEMS data were censored for known device non-use at least once in 32 participants (26.4%), with the censored data accounting for 4.6% of all monthly MEMS measurements. Median adherence levels for all measurement methods did not differ between children who were already taking ART and those who initiated ART at enrollment (Wilcoxon signed rank tests, p = 0.18–0.79), and median adherence did not change significantly over time (Wilcoxon signed rank test, p = 0.35). Median participant adherence clustered between 90% and 100% by all measures (Table 3); however, there were differences in the distribution of adherence for each measure. There were significantly more participants with <90% adherence by MEMS than three-day recall (29.6% versus 3.3%; p = <0.001). Median adherence among the ten children who left the study was 100.0% (IQR 100.0–100.0%) by three-day recall, 97.5% (IQR 93.8–100.0%) by 30-day VAS, 81.5% (IQR 80.8–85.9%) by unannounced pill counts/liquid formulation weights, and 84.6% (IQR 72.6–91.3%) by MEMS prior to disenrollment.

**Table 2:** Median adherence for the cohort by multiple measures.

	N	Median
3-day recall	119	100.0 (99.5–100.0)
30-day VAS	120	97.4 (93.0–100.0)
Unannounced pill counts/liquid formulation weights	117	97.3 (92.0–100.3)
MEMS	115	96.3 (88.5–99.1)

The interquartile range is shown in parentheses.

VAS  =  visual analog scale.

**Table 3:** Distribution of median adherence by participant using multiple measures.

	3-day recall		30-day VAS		Unannounced pill counts/liquid formulation weights		MEMS	
Median adherence (%)	N	%	N	%	N	%	N	%
100	87	73.1	31	25.8	33	28.1	23	20.0
≥95–<100	19	16.0	51	42.5	39	33.3	41	35.7
≥90–<95	9	7.6	23	19.2	23	19.7	17	14.8
≥80–<90	3	2.5	13	10.8	19	16.2	24	20.9
<80	1	0.8	2	1.7	3	2.6	10	8.7

VAS  =  visual analog scale.

Despite the high median values, interruptions in adherence were commonly detected by MEMS data. Sixty-nine children (57.0%) had periods in which their MEMS cap was not opened for ≥48 hours with a median of two such periods per child (IQR 1–5). The duration of these periods lasted a median of 106 hours (IQR 60–204). Analyzing children with both MEMS data and HIV RNA levels available at 12 months, 17 (44.7%) children with ≥48 hour interruptions and 12 (27.9%) children with no ≥48 hour interruptions had detectable HIV RNA. These levels of detectable HIV RNA were statistically similar, although a trend toward an association between ≥48-hour interruptions and detectable HIV RNA was seen (*X* ^2^ 2.5, p = 0.11).

### Correlation of Mean Adherence Measures and Log HIV RNA

Spearman correlations among all measures of adherence and log HIV RNA are shown in Table 4. Significant correlations were seen between the two subjective measures of mean adherence (three-day recall and VAS, r = 0.39, p<0.0001), as well as between MEMS and all other adherence measures (r = 0.24 with three-day recall, p = 0.009; r = 0.19 with VAS, p = 0.04; r = 0.41 with unannounced pill counts/liquid formulation weights, p<0.0001) over the one year study period. When comparing log HIV RNA with adherence in the month prior to the HIV RNA measurements, a statistically significant association was seen only with MEMS and log HIV RNA at the six-month time point (r = −0.25, p = 0.04). Log HIV RNA at six months was also significantly correlated with log HIV RNA at 12 months (r = 0.60, p<0.0001).

**Table 4:** Correlation among adherence measures and with HIV RNA.

	3-day recall	30-day VAS	Unannouncedpill counts/liquid formulation weights	MEMS	Log HIV RNA at 6 months	Log HIV RNA at 12 months
Mean value	98.7%	95.7%	96.1%	93.1%	–	–
3-day recall	–	**r = 0.39**	r = 0.05	**r = 0.24**	r = 0.12	r = 0.15
		**p<0.0001**	p = 0.60	**p = 0.009**	p = 0.35	p = 0.21
		N = 119	N = 117	N = 115	N = 73	N = 80
30-day VAS	–	–	r = −0.04	**r = 0.19**	r = −0.09	r = 0.05
			p = 0.64	**p = 0.04**	p = 0.49	p = 0.67
			N = 117	N = 115	N = 73	N = 80
Unannounced pill count/liquid formulation weights	–	–	–	**r = 0.41**	r = 0.14	r = 0.07
				**p<0.0001**	p = 0.28	p = 0.58
				N = 114	N = 72	N = 80
MEMS	–	–	–	–	**r = −0.25**	r = 0.00
					**p = 0.04**	p = 0.99
					N = 73	N = 80
Log HIV RNA 6 months	–	–	–	–	–	**r = 0.60**
						**p<0.0001**
						N = 70
Log HIV RNA 12 months	–	–	–	–	–	–

Adherence measures are compared with each other as means for all participants over the duration of the study. Comparisons between mean adherence measures and log HIV RNA reflect adherence in the month prior to the HIV RNA measurement. The first set of values in each box indicates the Spearman correlation coefficient (r).

VAS  =  visual analog scale.

Bold indicates p<0.05.

### Predictors of Adherence

MEMS adherence data was used as the outcome for the primary adherence analysis because it is an objective measure and significantly correlated with log HIV RNA. Table 5 presents the multivariable correlates of <90% MEMS adherence, including all variables with p<0.10 on univariable analysis (N = 81 due to missing values). Hospitalization of the child in the past three months (adjusted odd’s ratio [AOR] 3.0, 95% confidence interval [CI] 1.6–5.5; p = 0.001), any use of liquid formulations (AOR 1.4, 95% CI 1.0–2.0, p = 0.04), and caregiver’s use of alcohol (AOR 3.1, 95% CI 1.8–5.2; p<0.0001) were associated with <90% MEMS adherence. The child’s use of co-trimoxazole (AOR 0.5, 95% CI 0.4–0.9; p = 0.009), caregiver use of ART (AOR 0.6, 95% CI 0.4–0.9; p = 0.03), possible caregiver depression (AOR 0.6, 95% CI 0.4–0.8; p = 0.0001), and caregiver feeling ashamed of the child’s HIV status (AOR 0.5, 95% CI 0.3–0.6; p<0.0001) were protective against <90% MEMS adherence.

**Table 5:** Predictors of <90% MEMS adherence included in the multivariable model.

	Univariable	Multivariable
**Child characteristics**		
Age >5 years	0.9 (0.7–1.1; p = 0.37)	0.9 (0.7–1.3; p = 0.58)
Taking ART at enrollment	**0.6 (0.4–0.8; p = 0.001)**	1.0 (0.6–1.6; p = 0.89)
Enrollment CD4 percentage	**0.98 (0.98–0.99; p<0.0001)**	1.01 (0.99–1.02; p = 0.41)
Prior use of co-trimoxazole	0.9 (0.6–1.2; p = 0.36)	**0.5 (0.4–0.9; p = 0.009)**
Prior hospitalizations	**1.8 (1.1–2.8; p = 0.02)**	**3.0 (1.6–5.5; p = 0.001)**
**Regimen characteristics**		
Change in drug formulation	0.9 (0.7–1.3; p = 0.65)	1.5 (0.9–2.4; p = 0.08)
Any use of liquid formulations	**1.5 (1.2–1.9; p = 0.0004)**	**1.4 (1.0–2.0; p = 0.04)**
**Caregiver characteristics**		
Use of ART	0.9 (0.7–1.1; p = 0.20)	**0.6 (0.4–0.9; p = 0.03)**
Any alcohol use	**2.5 (1.7–3.7; p<0.0001)**	**3.1 (1.8–5.2; p<0.0001)**
Possible depression	**0.6 (0.5–0.8; p = 0.0001)**	**0.6 (0.4–0.8; p = 0.001)**
Ashamed of child’s HIV status	**0.8 (0.6–0.95; p = 0.02)**	**0.5 (0.3–0.6; p<0.0001)**
**Household/community characteristics**		
Place to keep ART	**0.4 (0.2–0.8; p = 0.009)**	*
Minutes to travel to clinic	1.0 (1.0–1.0; p = 0.19)	1.00 (1.00–1.01; p = 0.99)
Cost to travel to clinic	1.0 (1.0–1.0; p = 0.32)	1.00 (1.00–1.01; p = 0.13)

Values indicate odds ratios with 95% confidence intervals in parentheses.

* Could not be estimated in the multivariable model due to inadequate variation in values.

Bold indicates p<0.05.

Table 6 presents the multivariable correlates of the presence of ≥48-hour interruptions in MEMS adherence data, including all variables with p<0.10 on univariable analysis (N = 82 due to missing values). Change in drug manufacturer (AOR 4.1, 95% CI 1.5–11.5; p = 0.009) and any caregiver alcohol use (AOR 5.5, 95% CI 2.8–10.7; p<0.0001) were associated with the presence of ≥48-hour interruptions by MEMS, while use of second line ART (AOR 0.3, 95% CI 0.1–0.99, p = 0.049) and increasing household asset index (AOR 0.7; 95% CI 0.6–0.9; p<0.0007) were protective against ≥48-hour interruptions.

**Table 6:** Predictors of ≥48-hour interruptions MEMS adherence included in the multivariable model.

	Univariable	Multivariable
**Child characteristics**		
Age (one year increase)	1.1 (0.99–1.2; p = 0.07)	1.03 (0.9–1.2; p = 0.66)
Enrollment CD4 percentage	**0.9996 (0.9993–0.9999; p = 0.02)**	1.0003 (0.9998–1.0007; p = 0.29)
Attending school	**0.6 (0.4–0.9; p = 0.02)**	1.1 (0.6–2.0; p = 0.77)
**Regimen characteristics**		
Change in drug manufacturer	0.98 (0.5–1.8; p = 0.96)	**4.1 (1.5–11.5; p = 0.009)**
Any use of liquid formulations	**1.7 (1.2–2.6; p = 0.007)**	1.7 (0.97–3.1; p = 0.06)
Use of second line ART	**0.3 (0.1–0.96; p = 0.04)**	**0.3 (0.1–0.99; p = 0.049)**
**Caregiver characteristics**		
Greater than primary school	**0.4 (0.3–0.7; p = 0.001)**	0.6 (0.3–1.1; p = 0.13)
Employed	**0.6 (0.4–0.9; p = 0.006)**	0.8 (0.5–1.2; 0.26)
Any alcohol use	**4.9 (3.1–7.9; p<0.0001)**	**5.5 (2.8–10.7; p<0.0001)**
**Household/community characteristics**		
Place to keep ART	**0.2 (0.1–0.4; p<0.0001)**	0.3 (0.1–1.01; p = 0.05)
Minutes to travel to clinic	**0.993 (0.986–0.999; p = 0.02)**	1.004 (0.99–1.01; p = 0.41)
Disclosure to the community	**0.6 (0.4–0.8; p = 0.01)**	0.6 (0.4–1.1; p = 0.10)
Asset index	**0.7 (0.6–0.8; p<0.0001)**	**0.7 (0.6–0.9; p = 0.0007)**
# people in the household	**0.9 (0.8–0.95; p = 0.002)**	1.01 (0.9–1.1; p = 0.90)
# other children in the household	**0.9 (0.8–0.98; p = 0.02)**	0.9 (0.8–1.1; p = 0.41)
Having to sell property	**1.6 (1.1–2.4; p = 0.03)**	1.3 (0.8–2.3; p = 0.28)

Values indicate odds ratios with 95% confidence intervals in parentheses.

* Could not be estimated in the multivariate model due to inadequate variation in values.

Bold indicates p<0.05.

## Discussion

Multiple subjective and objective measures reveal high median adherence among Ugandan children receiving ART through an HIV/AIDS clinic in a rural, regional referral hospital. However, the fact that 57% of the children have ≥48 hour interruptions, 36.3% have detectable HIV RNA, and 30% have <90% adherence by MEMS indicates that a better understanding of adherence and adherence interventions are needed.

This study identified multiple factors that may be important for adherence success in HIV-infected children in developing settings. First, several associations suggest that a well-established medication taking routine is protective against poor adherence (defined as <90% MEMS and/or the presence of ≥48-hour interruptions in MEMS), which is consistent with other pediatric studies of HIV [5], [32] and asthma treatment [33], [34]. Co-trimoxazole use may reflect the importance of children developing effective routines applicable to ART adherence by taking co-trimoxazole to prevent opportunistic and other infections in the months prior to starting ART. While not retained in the multivariable models, the protective effects of a place to keep ART and taking ART at enrollment also support the importance of an established medication taking routine. Although treatment fatigue is a potential concern for children as their experience with ART lengthens [18], no decrease in adherence was seen over the one year of follow up in this study. The protective effect of second line ART may also reflect the role of treatment experience, albeit likely after initial adherence challenges. Additionally, changes in the ARV manufacturer and use of liquid formulations were associated with poor adherence. While some drug changes involved fixed drug combinations, which have been shown to increase adherence [35], [36], others were due to the child’s growth and development, as well as partial stock outs. Liquid formulations were often used as a means of maintaining the child’s regimen. Regardless of the reasons behind these changes, the association with poor adherence suggests that caregivers may have had trouble understanding the change in their dosing routine.

Study findings additionally support for the role of the caregiver in establishing a medication taking routine. The protective effect of caregiver use of ART suggests that caregivers taking ART themselves may have established, effective means for adherence, which can then be passed on to the children. The association of caregiver alcohol use with poor adherence may indicate disruption of this routine when the caregiver is intoxicated. A similar association between caregiver alcohol use and poor adherence for the child was seen in a recent South African study [37]. Alcohol use is also a common cause of poor adherence among adults taking ART [38], and this effect likely transfers to the child’s adherence. Additionally, the protective effect of increasing household assets against adherence interruptions may reflect the caregivers’ ability to overcome structural challenges in obtaining medications.

Second, children whose caregivers had possible depression and were ashamed of their child’s HIV status had lower odds of poor adherence. These findings may initially appear counter intuitive. For example, previous studies in Uganda, Ethiopia, and the US suggest a negative correlation between depression and adherence [39], [40], [41]; however, a South African study found no such association [37]. The differences among these studies and the current study may be due to varying cultural factors and/or measurement of depression. Importantly, the Hopkins Checklist used in this study assesses depressive symptoms and has been validated with a grief syndrome in Rwanda [25]. It does not diagnose clinical depression (i.e. major depressive disorder) per se. Moreover, the role of depression in adherence behavior is complex, especially when considering the caregiver-child dyad. Factors, such as guilt in the setting of vertical transmission, may manifest as depression [18], rather than more typical symptoms like hopelessness and psychomotor retardation.

Among the caregivers in this study, possible depression and shame may actually reflect positive motivational factors for caregivers to promote ART adherence in their children. For example, the most commonly endorsed symptom, “worrying too much about things”. Worry, as well as shame, could reflect an underlying concern and motivation to prevent poor outcomes in their children. This hypothesis should not be interpreted as an endorsement of depression and shame; further qualitative study is needed to understand this complex issue.

Third, the study also found that hospitalization was associated with poor adherence, which likely reflect adherence challenges prior to participation in this study. Interestingly, another Ugandan study found the opposite association on a cross-sectional analysis, which it attributed to a motivation to get well [8]. The dissimilarity in these results may be due to inclusion of relatively healthier children in this study.

MEMS appeared to be the best measure of adherence in this study for several reasons. First, as an objective measure, MEMS captures presumed dosing whether it is provided by a caregiver or initiated by the child; subjective measures often fail to accurately reflect both sources of information [18]. Second, MEMS was significantly correlated with HIV RNA, consistent with other pediatric studies in Africa [5], [17]. Third, MEMS had the widest distribution of adherence, which suggests that it may be better able to identify individuals with incomplete adherence. Additionally, MEMS was a feasible measure despite the common use of liquid formulations. MEMS, however, only had a modest correlation with log HIV RNA at one time point, which may be explained either by high rates of missing HIV RNA data, gaps in adherence which occurred prior to monitoring, and/or relatively limited variation in adherence values. Additionally, due to their expense, MEMS is not a practical outside a research setting.

Other adherence measures had distinct shortcomings. Unannounced pill counts/liquid weights were resource intensive and not associated with HIV RNA, possibly due to the inability to accurately track how much medication was dispensed during refill visits. The subjective measures of three-day recall and 30-day VAS did not correlate with HIV RNA, likely because of the minimal variance and the overestimation common with these measures [42]. The challenges seen with each adherence measure support a previous study reporting inadequate methods for use in resource-limited settings [43] and underscore the need for new approaches.

The type of adherence estimate is also important for understanding the relationship between adherence and viral suppression, as well as predictors of adherence behavior. The children in this study appear to have had sufficiently high median adherence to suppress viral replication with most modern, potent regimens [30]. The high levels of detectable HIV RNA in children without ART prior to study enrollment, however, indicate that high median adherence estimates do not ensure viral suppression. Although ≥48-hour interruptions and detectable HIV RNA were not significantly correlated in this study, the trend toward a positive association suggests such a relationship may be seen in larger sample sizes. Unfortunately, drug resistance data were not available for this analysis to better characterize the relationship between adherence interruptions and detectable HIV RNA. Surveillance data from Botswana suggest background HIV drug resistance in the region was likely to be very low at the time of the study, although rates in Uganda are unknown [44]. Further study of adherence interruptions could be facilitated by real-time adherence monitoring and HIV RNA testing [45], which may yield better information on which individuals are at risk for treatment failure. Additionally, predictors of adherence behavior differed somewhat between median adherence and ≥48-hour interruptions, suggesting that the factors influencing overall adherence behavior and interruptions in that behavior may be distinct to some extent.

This study has several limitations. First, the generalizability of the study results may be limited by the use of a stable, relatively healthy patient population living within 20 kilometers of the clinic. The same logistic considerations that made data collection problematic for children living farther than 20 kilometers from the clinic might have also impeded them in returning for pill dispensation and other care. Additional research is needed to better understand populations lost from care and how best to promote their retention in care and adherence to therapy. Second, the many adherence measures used in this study may have affected the adherence behavior of the study participants (i.e. the Hawthorne effect). While possible, adherence behavior altered by MEMS monitoring has been shown to revert to baseline within several weeks [46], and this study lasted one year. Third, the sample size, especially when considering missing data, may not be adequate to fully analyze correlates of adherence. Fourth, the exact nature of the hospitalizations is not known and the impact on adherence is speculative. Finally, the some of the measurement scales were developed and validated in developed countries and may not fully apply to this setting.

In conclusion, this study indicates that children in a rural, sub-Saharan African setting appear to have adequate median adherence to ART for viral suppression. However, the considerable number of interruptions in adherence and a high degree of detectable HIV RNA raise concern about their adherence and the long-term treatment success in this population. Interventions geared toward establishing good medication routines and addressing underlying motivational factors for the caregivers, such as fear of poor outcomes, may be potential means to promote successful adherence to ART.

## Supporting Information

Protocol S1 Partners Human Research Committee Protocol Summary.
(DOC)
