# Supplementary material for: Multiple Measures Reveal Antiretroviral Adherence Successes and Challenges in HIV-Infected Ugandan Children
Source: PLoS One. 2012 May 9;7(5):e36737. doi: 10.1371/journal.pone.0036737 (PMC3348916; doi:10.1371/journal.pone.0036737)
Supplement: Protocol S1 — Partners Human Research Committee Protocol Summary. (DOC) [file pone.0036737.s001.doc]

# Answer all questions accurately and completely in order to provide the PHRC with the relevant information to assess the risk-benefit ratio for the study. Do not leave sections blank.

# PRINCIPAL/OVERALL INVESTIGATOR

Jessica Haberer, MD, MS

# PROTOCOL TITLE

Measurement of adherence to antiretroviral therapy in HIV-infected school-aged children in Uganda

# FUNDING

National Institute of Mental Health R21MH083306

# VERSION DATE

This study was originally approved by the University of California, San Francisco Committee on Human Research on February 28, 2008, as well as the Mbarara University of Science and Technology (MUST) Institutional Review Board on April 14, 2008. This is the first submission to the Massachusetts General Hospital Institutional Review Board.

### SPECIFIC AIMS

Concisely state the objectives of the study and the hypothesis being tested.

**Specific Aim 1:** Compare several measurements of adherence to ART with each other and HIV RNA levels. These measures include unannounced pill counts and liquid medication weights, self-reported adherence through questionnaires and interactive voice response, a pill bottle that determines how many times it has been opened, pharmacy dispensing records, and hair samples.

**Specific Aim 2:** Using all of these measurements, determine how well HIV-infected children aged 1 to 10 years in Mbarara, Uganda take their ART.

**Specific Aim 3**: See how feasibility it is to use cell phones to perform unannounced pill counts and liquid medication weights, as well as to obtain self-reported adherence through interactive voice response, in a resource-limited setting.

We will conduct this study within the theoretical framework of the information, motivation, and behavioral (IMB) skills model of adherence behavior. While we do not intend to determine if the IMB model is appropriate for the study population, we will collect data to explore the factors influencing a child’s adherence to ART and to better understand how existing concepts should be adapted for more extensive study in the future. We will also collect qualitative data to identify additional factors that may not be included in the IMB model.

I anticipate adherence will be lower in objective measures than subjective. Average adherence rates will be lower than the those among adults in the same setting, but higher than those of children in developed countries. I believe the cell phone pill counts will be feasible.

# BACKGROUND AND SIGNIFICANCE

Provide a brief paragraph summarizing prior experience important for understanding the proposed study and procedures.

Dr. David Bangsberg is the primary mentor for this research study, and his group has studied measurements of ART adherence in adult populations in both the United States and Uganda[1-4]. These measurements have described critical relationships between adherence and decreased viral levels in the blood, disease progression, and the development of resistance to ART[5-15]. Other researchers have found similar relationships in pediatric patients in developed settings[16]. Studies of adherence to ART among non-adolescent children in developed settings have used a range of adherence measurements, including various forms of self-report[17-21], pharmacy records[21-25], pill counts[20,25], and electronic monitoring[20,21,25]. We are aware of only three studies[20,21,26] comparing multiple measures. Comparison among all studies has been limited by the lack of common sample characteristics and adherence assessment methodologies. Estimates of high adherence range from around 50% to 89%. The few studies in developing settings published before this study was developed in 2007 measured adherence through self-report[27-29] and pill count[30-31]. Reports of high adherence range from 66% to 90%. Multiple measures were not compared with each other or virus levels in these studies. Recently, unannounced pill counts by cell phone were shown to be very similar to those obtained through unannounced home visits among adults in a resource-rich setting (interclass correlation 0.990-0.997, p<0.001)[32,33]. Another study in US adolescents used daily phone diaries, along with self-report interviews and a clinical nurse rating, to measure adherence[34].

See References at the end of the document.

###### RESEARCH DESIGN AND METHODS

Briefly describe study design and anticipated enrollment, i.e., number of subjects to be enrolled by researchers study-wide and by Partners researchers. Provide a brief summary of the eligibility criteria (for example, age range, gender, medical condition). Include any local site restrictions, for example, “Enrollment at Partners will be limited to adults although the sponsor’s protocol is open to both children and adults.”

Throughout the study, the regular clinical care team in the Children’s HIV/AIDS Care Clinic at the Mbarara University Teaching Hospital provides the children with routine care, treatment of opportunistic infections, cotrimoxazole prophylaxis, nutritional counseling and support, and ART. Pediatricians document the World Health Organization clinical stage, and phlebotomy for CD4 count and viral load testing is performed at zero, six, and twelve months as part of routine care, or in the study if not performed for any reason. A small aliquot of blood is stored at each follow up time point to use for repeat testing in the event of equipment failure. If the stored blood specimen is not used, it is available for ART resistance or immune function testing at a later time. A research assistant administers a socio-demographic/adherence questionnaire at baseline and every three months thereafter for all subjects. He or she also performs qualitative interviews on adherence at baseline and six months for selected subject-caregiver pairs. Over the course of the one-year study, a research assistant makes an unannounced home visit for all caregivers once monthly to determine pill counts or liquid weights. Self-reported adherence measurements, the number of openings of an electronic pill bottle, and a brief symptom evaluation are also collected during the home visits. Hair samples are collected every three months at the home visits as well. For the caregivers selected to participate in the cell phone-based pill counts and/or liquid weights, a research assistant makes an unannounced call to the caregivers on the same day as the home visits, during which the caregiver counts his or her child’s pills or weigh the child’s medication bottles. Interactive voice response calls for self-reported adherence data will be made once monthly to all caregivers with cell phones. Additionally, the research assistant administers process measures at the six- and twelve-month home visits to assess for stigma and confidentiality. Finally, pharmacy records are assessed for each subject once a month.

Inclusion criteria

- 1-10 years of age;
- HIV-infected;
- Receiving HAART through the Children’s HIV/AIDS Care Clinic according to national Ugandan treatment guidelines:
  - - - CD4 count < 200 cells/mm3 or 15%, age 5 years and older
      - CD4 count < 350 cells/mm3 or 15%, age 3-5 years
      - CD4 count < 750 cells/mm3 or 20%, age 1-2 years
      - Development of a WHO stage 3 or 4 illness [62], regardless of CD4 count or percentage
      - Development of a WHO stage 1 or 2 illness if CD4 < 15%;
- Resident in Mbarara district (i.e., living within 30 km or two-hour driving radius of the city) due to logistics of home visits (less than 10% of potential participants live outside this radius);
- Plan to stay within the Mbarara district for the next year; and
- Written and signed consent of the primary caregiver and verbal assent of the child.

Exclusion criteria

The only exclusion criteria is attendance at a boarding school, which typically requires being away from home for three months at a time and would create considerable logistical challenges for the subjects.

Briefly describe study procedures. Include any local site restrictions, for example, “Subjects enrolled at Partners will not participate in the pharmacokinetic portion of the study.” Describe study endpoints.

1. Baseline WHO clinical staging, CD4 count and viral load testing (done regardless of enrollment). If these lab tests are not available for ART naïve subjects within three months of enrollment, they are repeated as part of the study.
2. Baseline data collection on factors potentially influencing adherence
   1. For all subjects: one 60-90 minute session at home in which a research assistant administers a socio-demographic/adherence questionnaire to the caregiver and cognitive/behavioral tests to the child. Please see the attachment “Socio-demographic/adherence questionnaire” for details.

b. For 50 subjects (selected randomly within each year of age): an additional 30-minute qualitative interview. Please see the attachment “Qualitative Interview” for details.

1. Monthly adherence data collection at home for all subjects for the one-year course of the study, including the collection of hair samples every three months, if available.
2. Monthly adherence data collection via a cell phone for half of the subjects whose caregivers have cell phones (randomly selected at enrollment) for the one-year course of the study.
3. At three-month intervals, repeated administration of the socio-demographic/adherence questionnaire during a home visit. Please see the attachment “Socio-demographic/adherence questionnaire” for details.
4. At six months, clinical staging, CD4 count and viral load testing (done regardless of enrollment). Two milliliters of additional blood are drawn and stored for repeat testing in the event of equipment malfunction. If the CD4 count and viral load are not available for some reason, they are repeated as part of the study.
5. At six months, process measure questions to assess for stigma and disclosure, as well as re-administration of the qualitative interview (for the same sample selected for the baseline qualitative interview). Please see the attachment “Qualitative interview” for details.
6. At twelve months, clinical staging, CD4 count and viral load testing (done regardless of enrollment). Two milliliters of additional blood are drawn and stored for repeat testing in the event of equipment malfunction. If the CD4 count and viral load are not available for some reason, they will be repeated as part of the study.
7. At twelve months, process measure questions to assess for stigma and disclosure. Please see the attachment “Process Measures” for details.

For studies involving treatment or diagnosis, provide information about standard of care at Partners (e.g., BWH, MGH) and indicate how the study procedures differ from standard care. Provide information on available alternative treatments, procedures, or methods of diagnosis.

N/A

Describe how risks to subjects are minimized, for example, by using procedures which are consistent with sound research design and which do not unnecessarily expose subjects to risk or by using procedures already being performed on the subject for diagnostic or treatment purposes.

The most significant risk to patients is the potential for loss of privacy and disclosure of HIV status, and we take the following steps to protect against these risks. First, all investigators and staff associated with the study have undergone human subjects training with the National Institutes of Health modules prior to the implementation of the study and sign a pledge of confidentiality. Second, we have set up several mechanisms to ensure confidentiality of subjects. All interviews occur in a private space. Contact with health care providers and other health services agencies only occurs with prior written consent from the subject’s caregiver. The nature of any information that might be disclosed is included as part of this consent. Subjects are also informed of their right to discontinue participation in the study at any time. All paper files are stored in locked file cabinets and electronic files stored in password-protected files. Furthermore, both paper and electronic files, including HIV viral load, and CD4 cell count laboratory reports, are identified only by the subject’s identification (ID) number. The investigators and project coordinator retain identifying information linking subjects to their study ID number off site in a locked cabinet only accessible to them. To protect against HIV disclosure, our research assistants are members of the local community and only approach subjects on foot. Most visits therefore go unnoticed. We also collect information regarding times that the subject and/or caregiver prefer not to have a study visit and encourage them to provide the research assistant with a code word or sign to indicate that the visit should not take place, if concerns about disclosure of the subject’s HIV status arise. Moreover, we are collecting process measures regarding the intrusiveness and convenience of the home-based and cell phone-based pill counts at the six month and twelve month time points during the study. The institutional review boards at Partners’ and the Mbarara University of Science and Technology will be notified of any breaches in confidentiality, HIV disclosure, or other adverse events attributable to this study. To mitigate any concerns about witchcraft regarding the hair samples, we carefully explain to the caregivers during the consent process why we are collecting the hair and how it will be used. We also clearly give subjects and their caregivers the option of not providing hair samples at any point in the study. Finally, we keep the number of blood draws to a minimum by using the results of routine clinical labs whenever possible. We advise the caregivers that the stored blood samples will only be marked with an ID number, not the child’s name, to protect confidentiality. Any additional tests on unused specimens will only relate to HIV treatment, and the results will be made available to the caregivers and the clinicians. Moreover, the caregivers are informed that they may have the stored blood samples destroyed at any time.

Describe explicitly the methods for ensuring the safety of subjects. Provide objective criteria for removing a subject from the study, for example, objective criteria for worsening disease/lack of improvement and/or unacceptable adverse events. The inclusion of objective drop criteria is especially important in studies designed with placebo control groups.

We take all necessary steps to ensure the safety of our subjects, as well as the validity and integrity of the data. Please see the preceding paragraph for details relating to confidentiality. The investigators review confidentiality policies and procedures with all new staff and annually with current staff. The local principal investigators, Drs. Kiwanuka and Nansera, monitor these confidentiality practices on a monthly basis and communicate their findings to Dr. Haberer. She personally monitors the confidentiality practices on two or more trips to Mbarara annually. Additionally, our study does not impose any health risk greater than is normally associated with taking ART in Uganda.

We would remove a subject from the study if he or she moves beyond the 30 km radius of the clinic, or if he or she attends boaring school, thus violating the inclusion or exclusion criteria for the study. We would also remove a subject if his or her caregiver changes and does not wish to continue participation in the study.

# FORESEEABLE RISKS AND DISCOMFORTS

Provide a brief description of any foreseeable risks and discomforts to subjects. Include those related to drugs/devices/procedures being studied and/or administered/performed solely for research purposes. In addition, include psychosocial risks, and risks related to privacy and confidentiality. When applicable, describe risks to a developing fetus or nursing infant.

Some subjects may have slightly increased discomfort from the additional blood drawn, and caregivers may be uncomfortable about the idea of stored blood samples for future testing. Subjects will not face any health risk greater than what they normally would when taking ART in Uganda, and we are not studying any new devices or procedures. The most significant risk to patients is the potential for loss of privacy and disclosure of HIV status. Some people may also be concerned about witchcraft with the use of hair samples.

**EXPECTED BENEFITS**

# Describe both the expected benefits to individual subjects participating in the research and the importance of the knowledge that may reasonably be expected to result from the study. Provide a brief, realistic summary of potential benefits to subjects, for example, “It is hoped that the treatment will result in a partial reduction in tumor size in at least 25% of the enrolled subjects.” Indicate how the results of the study will benefit future patients with the disease/condition being studied and/or society, e.g., through increased knowledge of human physiology or behavior, improved safety, or technological advances.

Caregivers for subjects may become more aware of the subject’s adherence problems by completing the structured interviews and partaking in the adherence assessments. They may also benefit from knowing that their participation may help others like them have better ART adherence in the future. Additionally, the banked blood increases the likelihood that the child will get viral load results every six months, regardless of problems running the sample. If additional tests are performed on the banked blood, we will attempt to contact the caregivers with the results. The caregiver may then elect to share this information with the child’s physician for possible improvement in care.

The vast majority of the 2.1 million children with HIV/AIDS live in resource-limited settings, and few have received ART. Fortunately, treatment programs for these children are becoming more available. Proper adherence is critical to optimizing the benefits of ART, and a better understanding of adherence rates and associated factors in Ugandan children receiving ART will allow for the development of tailored intervention efforts and medication adherence support programs.

**EQUITABLE SELECTION OF SUBJECTS**

The risks and benefits of the research must be fairly distributed among the populations that stand to benefit from it. No group of persons, for example, men, women, pregnant women, children, and minorities, should be categorically excluded from the research without a good scientific or ethical reason to do so. Please provide the basis for concluding that the study population is representative of the population that stands to potentially benefit from this research.

The nature of this study is limited to young, HIV-infected children taking ART in a developing setting. We chose not to include adults or older children because of the many differences in development and other factors potentially influencing adherence behavior. Pregnancy is not a relevant issue for the ages we are studying. Moreover, many other studies are looking or have looked at ART adherence in adults. We are conducting the study in one site, the Children’s HIV/AIDS Care Clinic in the Mbarara University Teaching Hospital, which is located in a developing, largely rural setting. We are approaching all HIV-infected children aged 1 to 10 years old who are receiving ART in this clinic. The population of Uganda is 95% Africa/ 5% Asian and 55% female/45% male. We are enrolling all children who meet the inclusion and exclusion criteria of the study and are not discriminating against any racial group or sex in our study site. We believe this study site is representative of many developing areas in sub-Saharan Africa given its low per capita income, use of free ART, and use of locally trained doctors. It should therefore be relevant for the 1.9 million children estimated to have HIV in this region.

When people who do not speak English are excluded from participation in the research, provide the scientific rationale for doing so. Individuals who do not speak English should not be denied participation in research simply because it is inconvenient to translate the consent form in different languages and to have an interpreter present.

This study is being conducted in rural Uganda, using the two languages spoken in this area (English and Runyankole).

For guidance, refer to the following Partners policy:

Obtaining and Documenting Informed Consent of Subjects who do not Speak English

[**http://healthcare.partners.org/phsirb/nonengco.htm**](http://healthcare.partners.org/phsirb/nonengco.htm)

**RECRUITMENT PROCEDURES**

Explain in detail the specific methodology that will be used to recruit subjects. Specifically address how, when, where and by whom subjects will be identified and approached about participation. Include any specific recruitment methods used to enhance recruitment of women and minorities.

A research assistant identifies potential subjects by chart review in the Children’s HIV/AIDS Care Clinic. Children and their caregivers are invited to enroll in this study after the attending doctor in the clinic confirms the child’s eligibility. We are approaching all children in the clinic regardless of sex or race.

Provide details of remuneration, when applicable. Even when subjects may derive medical benefit from participation, it is often the case that extra hospital visits, meals at the hospital, parking fees or other inconveniences will result in additional out-of-pocket expenses related to study participation. Investigators may wish to consider providing reimbursement for such expenses when funding is available

Subjects and caregivers are given 1,000 – 5,000 Ugandan Shillings (approximately US $0.60 - $3.00) per person for reimbursement of transportation costs associated with each of the three clinic visits (zero, six, and twelve months) at the time of the visit. The amount given depends on the distance traveled. Additionally, research assistants provide subjects and caregivers with either a bar of soap or a pound of sugar at each home visit (estimated value $0.60). These payment have been used in other studies conducted at Mbarara University of Science and Technology and has been well received and approved by its Institutional Review Board.

For guidance, refer to the following Partners policies:

Recruitment of Research Subjects

[**http://healthcare.partners.org/phsirb/recruit.htm**](http://healthcare.partners.org/phsirb/recruit.htm)

Guidelines for Advertisements for Recruiting Subjects

[**http://healthcare.partners.org/phsirb/advert.htm**](http://healthcare.partners.org/phsirb/advert.htm)

Remuneration for Research Subjects

[**http://healthcare.partners.org/phsirb/remun.htm**](http://healthcare.partners.org/phsirb/remun.htm)

#### CONSENT PROCEDURES

Explain in detail how, when, where, and by whom consent is obtained, and the timing of consent (i.e., how long subjects will be given to consider participation). For most studies involving more than minimal risk and all studies involving investigational drugs/devices, a licensed physician investigator must obtain informed consent. When subjects are to be enrolled from among the investigators’ own patients, describe how the potential for coercion will be avoided.

A research assistant obtains informed consent from the primary caretaker and verbal assent from the child (if age 7 or older), after a detailed explanation of the objectives and procedures of the study. Many of the eligible children have non-biologic caregivers, and there is no standard legal system of guardianship in Uganda at this time. We therefore ask caregivers to attest of their status and document it by signing a Parent/Guardian Declaration and Authorized Attendants form. Caregivers may also change during the course of the study, in which case a research assistant repeats the informed consent process. If the caregiver does not give consent or the child does not give verbal assent, neither individual will be enrolled in the study. All verbal and written information is delivered in English or the patient’s local vernacular (Runyankole), according to his or her preference.

NOTE: When subjects are unable to give consent due to age (minors) or impaired decision-making capacity, complete the forms for Research Involving Children as Subjects of Research and/or Research Involving Individuals with Impaired Decision-making Capacity, available on the New Submissions page on the PHRC website:

**[http://healthcare.partners.org/phsirb/newapp.htm#Newapp](http://healthcare.partners.org/phsirb/newapp.htm#Newapp)**

For guidance, refer to the following Partners policy:

Informed Consent of Research Subjects

[**http://healthcare.partners.org/phsirb/infcons.htm**](http://healthcare.partners.org/phsirb/infcons.htm)

## DATA AND SAFETY MONITORING

Describe the plan for monitoring the data to ensure the safety of subjects. The plan should include a brief description of (1) the safety and/or efficacy data that will be reviewed; (2) the planned frequency of review; and (3) who will be responsible for this review and for determining whether the research should be altered or stopped. Include a brief description of any stopping rules for the study, when appropriate. Depending upon the risk, size and complexity of the study, the investigator, an expert group, an independent Data and Safety Monitoring Board (DSMB) or others might be assigned primary responsibility for this monitoring activity.

NOTE: Regardless of data and safety monitoring plans by the sponsor or others, the principal investigator is ultimately responsible for protecting the rights, safety, and welfare of subjects under his/her care.

Although we do not have a formal Data Safety Monitoring Board or Data Monitoring Committee, we take all necessary steps to ensure the safety of our subjects, as well as the validity and integrity of the data. Please see the sections on risk and safety above for details. Additionally, data management team already exists at the Ugandan Research Initiative in Mbarara for Dr. Bangsberg’s adult adherence studies and assists with this study as well. A dedicated and trained data entry technician enters data on subject registration, enrollment, and informed consent, as well as socio-demographic/adherence questionnaires, into an Access database per a standard protocol, and the project coordinator and/or research assistants verify key texts. Adherence measurement data is similarly entered into a separate Access database that includes algorithms to automatically check for missing, out-of-range, or inconsistent values. This relational database links laboratory, adherence, treatment history, drug resistance, and death records by a unique subject identifier and date. The data is available on a secure web site, and aberrant entries are reported immediately to data entry technician for reconciliation. The project coordinator and/or investigators check all reports at least monthly for accuracy. An audit trail is created for all data changes. Databases are maintained in an NT-operating system in a password-protected partitioned drive, and backed up daily and quarterly (24G NT Backup).

The investigators review all safety and data management policies and procedures with new staff and annually with current staff. Drs. Kiwanuka and Nansera monitor these practices on a monthly basis and communicate their findings to Dr. Haberer. She personally monitors them on two or more trips to Mbarara annually. The investigators will determine if the research should be altered or stopped. We have not developed formal stopping rules, as the nature of the study is observational. However, extremely high levels of low adherence (i.e. over 50% of subjects with less than 80% adherence) would prompt such discussions, as these children would be at high risk for treatment failure and systemic problems in care delivery would be likely.

Describe the plan to be followed by the Principal Investigator/study staff for review of adverse events experienced by subjects under his/her care, and when applicable, for review of sponsor safety reports and DSMB reports. Describe the plan for reporting adverse events to the sponsor and the Partners’ IRB and, when applicable, for submitting sponsor safety reports and DSMB reports to the Partners’ IRBs. When the investigator is also the sponsor of the IND/IDE, include the plan for reporting of adverse events to the FDA and, when applicable, to investigators at other sites.

NOTE: In addition to the adverse event reporting requirements of the sponsor, the principal investigator must follow the Partners Human Research Committee guidelines for Adverse Event Reporting

Because we are collecting data on caregiver-child pairs, we may learn of child abuse. We will report any problems to the MUST Institutional Review Board, who would then alert the authorities according to Ugandan law. Additionally, if we identify problems with adherence, we remind the caregiver of the adherence counseling available in the Children’s HIV/AIDS Care Clinic. This counseling represents the existing standard of care. We therefore think it is the ethical response and will not bias our study results. Finally, we refer all subjects to the Children’s HIV/AIDS Care Clinic for medical care if illness is detected. All reports will be made as soon as possible, typically within 48 hours of detection or emergently if indicated. We are not using any devices or experimental drugs.

## MONITORING AND QUALITY ASSURANCE

Describe the plan to be followed by the principal investigator/study staff to monitor and assure the validity and integrity of the data and adherence to the IRB-approved protocol. Specify who will be responsible for monitoring, and the planned frequency of monitoring. For example, specify who will review the accuracy and completeness of case report form entries, source documents, and informed consent.

NOTE: Regardless of monitoring plans by the sponsor or others, the principal investigator is ultimately responsible for ensuring that the study is conducted at his/her investigative site in accordance with the IRB-approved protocol, and applicable regulations and requirements of the IRB.

Please see the section above on Data and Safety Monitoring for details of our data entry and review policies. In sum, the project coordinator and/or research assistants verify key data on subject registration, enrollment, and informed consent, as well as data from the socio-demographic/adherence questionnaires in one Access database. This information is reviewed reviewed within one week of entry. The project coordinator additionally reviews all subject registration, enrollment, and informed consent records on a monthly basis. The second Access database for adherence measurement data contains an algorithm that automatically and immediately reviews data for missing, out-of-range, or inconsistent values. The project coordinator and/or investigators check this database at least monthly for accuracy using source documents. The audits consist of at least 10% of randomly selected subjects.

For guidance, refer to the following Partners policies:

##### Data and Safety Monitoring Plans and Quality Assurance

[**http://healthcare.partners.org/phsirb/datasafe.htm**](http://healthcare.partners.org/phsirb/datasafe.htm)

Adverse Event Reporting Guidelines

[**http://healthcare.partners.org/phsirb/adverse_events.htm**](http://healthcare.partners.org/phsirb/adverse_events.htm%0C)

# PRIVACY AND CONFIDENTIALITY

Describe methods used to protect the privacy of subjects and maintain confidentiality of data collected. This typically includes such practices as substituting codes for names and/or medical record numbers; removing face sheets or other identifiers from completed surveys/questionnaires; proper disposal of printed computer data; limited access to study data; use of password-protected computer databases; training for research staff on the importance of confidentiality of data, and storing research records in a secure location.

NOTE: Additional measures, such as obtaining a Certificate of Confidentiality, should be considered and are strongly encouraged when the research involves the collection of sensitive data, such as sexual, criminal or illegal behaviors.

The privacy of subjects and the confidentiality of collected data are protected by assigning each subject a unique ID number (i.e. MBB4xxx). The paper files linking the subject name to the ID number are stored in locked file cabinets and electronic files stored in password-protected files, which are only available to the investigators and project coordinator. All collected data (i.e. socio-demographic/adherence questionnaire, adherence measurements, laboratory data) are only identified by this subject ID number. All paper files are stored in locked file cabinets and electronic files stored in password-protected files. All staff are required to take annual training in confidentiality and research ethics. We are very concerned about the confidentiality of our data; however, we will not obtain a Certificate of Confidentiality. The study is conducted entirely in Uganda, and such protection does not apply in a foreign country.

SENDING SPECIMENS/DATA TO RESEARCH COLLABORATORS OUTSIDE PARTNERS

Specimens or data collected by Partners investigators will be sent to research collaborators outside Partners, indicate to whom specimens/data will be sent, what information will be sent, and whether the specimens/data will contain identifiers that could be used by the outside collaborators to link the specimens/data to individual subjects.

Hair samples will be sent to Dr. Monica Gandhi at the University of California, San Francisco for drug levels. The samples will only be identified with a unique ID number, which will not enable linkage of the specimens to individual subjects.

Specifically address whether specimens/data will be stored at collaborating sites outside Partners for future use not described in the protocol. Include whether subjects can withdraw their specimens/data, and how they would do so. When appropriate, submit documentation of IRB approval from the recipient institution.

Approximately 2 ml of blood from each blood draw will be stored at the Mbarara University of Science and Technology. This blood will be used for viral load tests in the event the Children’s HIV/AIDS Clinic can not determine a viral load (i.e. equipment malfunction, sample spilled). If the stored blood specimens are not used for repeat viral load testing, we will store the blood for possible ART resistance tests and/or immune cell function tests at a later time. If these tests are run, we will make the results available to the caregivers and subjects, who may share this information with their clinicians. The specimens will be labeled with the subject ID number and date of collection only to protect the subject’s confidentiality. Caregivers are informed that the specimens can be removed and destroyed at any time by notifying the study investigators or project coordinator. Please see the submitted IRB approval from the Mbarara University of Science and Technology.

# RECEIVING SPECIMENS/DATA FROM RESEARCH COLLABORATORS OUTSIDE PARTNERS

When specimens or data collected by research collaborators outside Partners will be sent to Partners investigators, indicate from where the specimens/data will be obtained and whether the specimens/data will contain identifiers that could be used by Partners investigators to link the specimens/data to individual subjects. When appropriate, submit documentation of IRB approval and a copy of the IRB-approved consent form from the institution where the specimens/data were collected.

Data for the study will be collected through the Mbarara University of Science and Technology/Mbarara University Teaching Hospital. Data will contain the subject’s name, date of birth, residence, and laboratory values. This information will be kept confidential and only available to the study investigators and project coordinator, as described above. The IRB approval from MUST and approved consent form is submitted. A renewal will be filed shortly.

**References**

1. Bangsberg DR, Hecht FM, Charlebois ED, Zolopa AR, Holodniy M, Sheiner L, Bamberger JD, Chesney MA, Moss A. Adherence to protease inhibitors, HIV-1 viral load, and development of drug resistance in an indigent population. AIDS 2000;14(4):357-66.

2. Oyugi JH, Byakika-Tusiime J, Charlebois ED, Kityo C, Mugerwa R, Mugyenyi P, Bangsberg DR. Multiple validated measures of adherence indicate high levels of adherence to generic HIV antiretroviral therapy in a resource-limited setting. Journal of Acquired Immune Deficiency Syndromes 2004;36(5):1100-2.

3. Bangsberg D, Hecht F, Charlebois E, Chesney M, Moss A. Comparing objectives measures of adherence to HIV antiretroviral therapy: electronic medication monitors and unannounced pill counts. AIDS and Behavior 2001;5(3):275-81.

4. Giordano T, Guzman D, Clark R, Charlebois E, Bangsberg D. Measuring adherence to antiretroviral therapy in a diverse population using a visual analogue scale. HIV Clinical Trials 2004;5(2):74-9.

5. Bangsberg DR, Perry S, Charlebois ED, Clark RA, Roberston M, Zolopa AR, Moss A. Non-adherence to highly active antiretroviral therapy predicts progression to AIDS. AIDS 2001;15(9):1181-3.

6. Hogg R, Heath K, Bangsberg D, Yip B, Press N, O'Shaughnessy M, Montaner J, Incomplete adherence to triple combination therapy is predictive of mortality at baseline and after one year of follow-up. AIDS 2002;16(7):1051-8.

7. Byakika-Tusiime J, Oyugi JH, Tumwikirize WA, Katabira ET, Mugyenyi PN, Bangsberg DR. Adherence to HIV antiretroviral therapy in HIV+ Ugandan patients purchasing therapy. International Journal of STDs and AIDS 2005;6(1):38-41.

8. Bangsberg DR, Acosta E, Gupta R, Guzman D, Riley ED, Harrigan PR, Parkin N, Deeks S. Differences in protease and non-nucleoside reverse transcriptase inhibitor adherence-resistance relationships are explained by virologic fitness. AIDS 2006 Jan 9;20(2):223-31.

9. Bangsberg DR, Weiser S, Guzman D, Riley ED. 95% adherence is not necessary for viral suppression to less than 400 copies/mL in the majority of individuals on NNRTI regimens. 12th Conference on Retroviruses and Opportunistic Infections (CROI) 2005 in Boston. Paper #616.

10. Bangsberg DR, Moss AR, Deeks SG. Paradoxes of HIV antiretroviral adherence and drug resistance. Journal of Antimicrobial Chemotherapy, 2004;53(5):696-9.

11. Bangsberg DR, Porco TC, Kagay C, Charlebois ED, Deeks SG, Guzman D, Clark R, Moss A. Modeling the HIV protease inhibitor adherence-resistance curve by use of empirically derived estimates. Journal of Infectious Diseases 2004;190(1):162-5.

12. Bangsberg D, Gupta R, Harrigan P, Guzman D, Riley E, Clark R, Deeks S. Low levels of adherence confers greater risk for non-nucleoside reverse transcriptase inhibitor resistance than protease-inhibitor resistance. 13th International HIV Drug Resistance Workshop 2004 in the Canary Islands, Spain: Antiviral Therapy 2004;9:S179.

13. Bangsberg D, Charlebois E, Grant R, Holodiny M, Perry S, Nugent J, Zolopa A, Moss A. High levels of adherence do not prevent the accumulation of drug resistance mutations to HIV antiretroviral therapy. AIDS 2003;17:1925-32.

14. Bangsberg DR, Ware N, Simoni J. Adherence without access to antiretroviral therapy in sub-Saharan Africa. AIDS 2006 Jan 2;20(1):140-1.

15. Kagay CR, Porco TC, Liechty CA, Charlebois E, Clark R, Guzman D, Moss AR, Bangsberg DR. Modeling the impact of modified directly observed antiretroviral therapy on HIV suppression and resistance, disease progression, and death. Clinical Infectious Diseases 2004;38 Suppl 5:S414-20.

16. Van Dyke RB, Lee S, Johnson GM, Wiznia A, Mohan K, Stanley K, Morse EV, Krogstad PA, Nachman S; Pediatric AIDS Clinical Trials Group Adherence Subcommittee Pediatric AIDS Clinical Trials Group 377 Study Team. Reported adherence as a determinant of response to highly active antiretroviral therapy in children who have human immunodeficiency virus infection. Pediatrics 2002;109(4):e61.

17. Reddington C, Cohen J, Baldillo A, Toye M, Smith D, Kneut C, Demaria A, Bertolli J, Hsu HW. Adherence to medication regimens among children with human immunodeficiency virus infection. Pediatric Infectious Disease Journal 2000;19(12):1148-53.

18. Mellins CA, Brackis-Cott E, Dolezal C, Abrams EJ. The role of psychosocial and family factors in adherence to antiretroviral treatment in human immunodeficiency virus-infected children. Pediatric Infectious Disease Journal 2004;23(11):1035-41.

19. Naar-King S, Arfken C, Frey M, Harris M, Secord E, Ellis D. Psychosocial factors and treatment adherence in paediatric HIV/AIDS. AIDS Care 2006;18(6):621-8.

20. Steele RG, Anderson B, Rindel B, Dreyer ML, Perrin K, Christensen R, Tyc V, Flynn PM. Adherence to antiretroviral therapy among HIV-positive children: examination of the role of caregiver health beliefs. AIDS Care 2001;13(5):617-29.

21. Farley JJ, Hines SE, Musk AE, Ferrus S, Tepper V. Assessment of adherence to antiviral therapy in HIV-infected children using the Medication Event Monitoring System, pharmacy refill, provider assessment, caregiver self-report, and appointment keeping. Journal of Acquired Immune Deficiency Syndromes 2003;33, 211-218.

22. Marhefka SL, Tepper VJ, Brown JL, Farley JJ. Caregiver psychosocial characteristics and children's adherence to antiretroviral therapy. AIDS Patient Care and STDS 2006;20(6):429-37.

23. Watson DC, Farley JJ. Efficacy of and adherence to highly active antiretroviral therapy in children infected with human immunodeficiency virus type 1. Pediatric Infectious Disease Journal 1999;18(8):682-9.

24. Katko E, Johnson GM, Fowler SL, Turner RB. Assessment of adherence with medications in Human Immunodeficiency Virus-infected children. Pediatric Infectious Disease Journal 2001;20(12):1174-1176.

25. Temple ME, Koranyi K, Nahata MC. The safety and antiviral effect of protease inhibitors in children. Pharmacotherapy 2001;21:287-294.

26. Martin S, Elliott-DeSorbo DK, Wolters PL, Toledo-Tamula MA, Roby G, Zeichner S, Wood LV. Patient, caregiver and regimen characteristics associated with adherence to highly active antiretroviral therapy among HIV-infected children and adolescents. Pediatric Infectious Disease

27. Bikaako-Kajura W, Luyirika E, Purcell DW, Downing J, Kaharuza F, Mermin J, Malamba S, Bunnell R. Disclosure of HIV status and adherence to daily drug regimens among HIV-infected children in Uganda. AIDS & Behavior 2006;10:S85-S93.

28. Nyandiko WM, Ayaya S, Nabakwe E, Tenge C, Sidle JE, Yiannoutsos CT, Musick B, Wools-Kaloustian K, Tierney WM. Outcomes of HIV-infected orphaned and non-oprhaned children on antiretroviral therapy in Western Kenya. Journal of Acquired Immune Deficiency Syndromes 2006; 43:418-25.

29. Elise A, France AM, Louise WM, Bata D, Francois R, Roger S, Philippe M. Assessment of adherence to highly active antiretroviral therapy in a cohort of African HIV-infected children in Abidjan, Cote d’Ivoire. Journal of Acquired Immune Deficiency Syndromes 2005;40(4):498-500.

30. Eley B, Nuttall J, Davies MA, Smith L, Cowburn C, Buys H, Hussey G. Initial experience of a public sector antiretroviral treatment programme for HIV-infected children and their infected parents. South African Medical Journal 2004;94(8):643-6.

31. Hansudewechakul, R, Jourdain, G, Plangraun, N, The Chiang Rai Paediatric Arv Team. A comprehensive programme to strengthen adherence to antiretroviral drug therapy and achieve virological control in HIV infected children in Thailand. Vulnerable Children and Youth Studies 2006;1(2):180-191.

32. Kalichman SC Amaral CM, Stearns H, White D, Flanagan J, Pope H, Cherry C, Cain D, Eaton L, Kalichman MO. Adherence to antiretroviral therapy assess by unannounced pill counts conducted by telephone. Journal of General Internal Medicine 2007;22(7):1003-6.

33. Kalichman SC, Amaral CM, Cherry C, Flanagan J, Pope H, Eaton L, Kalichman MO, Cain D, Detorio M, Caliendo A, Schinazi RF. Monitoring medication adherence by unannounced pill counts conducted by telephone: reliability and criterion-related validity. HIV Clin Trials. 2008 Sep-Oct;9(5):298-308.

34. Wiener L, Riekert K, Ryder C, Wood LV. Assessing medication adherence in adolescents with HIV when electronic monitoring is not feasible. AIDS Patient Care STDS. 2004;18(9):527-38.
